# Microplastics in the seminal microenvironment of boar semen: associations with sperm motility and antimicrobial susceptibility

**DOI:** 10.3389/fvets.2026.1847076

**Published:** 2026-05-26

**Authors:** Šarūnė Sorkytė, Steigvilė Byčenkienė, Ieva Uogintė, Sonata Pleskytė, Artūras Šiukščius, Marius Virgailis, Rita Šiugždinienė, Gintarė Vaičiulienė, Audronė Rekešiūtė, Neringa Sutkevičienė

**Affiliations:** 1 Animal Reproduction Laboratory, Faculty of Veterinary Medicine, Veterinary Academy, Lithuanian University of Health Sciences, Kaunas, Lithuania; 2 Department of Environmental Research, Center for Physical Sciences and Technology (FTMC), Vilnius, Lithuania; 3 Department of Animal Breeding and Reproduction, Institute of Animal Science, Veterinary Academy, Lithuanian University of Health Sciences, Baisogala, Lithuania; 4 Institute of Microbiology and Virology, Veterinary Academy, Lithuanian University of Health Sciences, Kaunas, Lithuania

**Keywords:** antibiotic susceptibility, boar semen, CASA, microplastics, semen microbiota

## Abstract

Microplastics (MPs) are increasingly recognized as environmental contaminants capable of interacting with biological systems. However, their presence in livestock reproductive fluids remains relatively unexplored. In swine artificial insemination, boar semen quality is a key determinant of reproductive efficiency, and spermatozoa are highly susceptible to physicochemical stressors present in their surrounding microenvironment. Particulate contaminants such as MPs may therefore represent an overlooked factor relevant to semen quality. The present study investigated the occurrence, polymer composition, and size distribution of MPs in undiluted boar semen using μFTIR spectroscopy. In addition, exploratory analyses were conducted to evaluate potential associations between MPs and semen motility parameters assessed using computer-assisted sperm analysis (CASA) system, as well as bacterial load and antimicrobial susceptibility profiles of bacteria isolates. MPs were detected in all analyzed semen samples (*n* = 12), with concentration median (min–max) 9.58 (0.48–19.00) MPs/ml. Considerable variability was observed between individual boars and between the two participating breeding facilities. Polyethylene was the most frequently identified polymer. Exploratory analyses revealed that higher concentrations of specific polymers, particularly polyethylene and polyester, were associated with differences in sperm velocity distribution, characterized by a lower proportion of rapidly motile spermatozoa and a higher proportion of spermatozoa with slow velocity. Although there was no association between MPs levels and total bacterial counts, limited statistically significant associations were identified between the antimicrobial susceptibility profiles of the most frequently isolated bacterial species and specific MPs polymer types. These findings suggest that MPs may be related to sperm function and bacteria–antibiotic interactions within the seminal microenvironment. This study provides, to our knowledge, the first evidence of MPs in boar semen and identifies MPs as a potential environmental contamination factor relevant to semen quality.

## 1 Introduction

The efficiency of swine artificial insemination depends on the consistent availability of high-quality boar semen, making routine semen quality assessment an essential component of reproductive management in commercial pig breeding programs (1). A single boar may contribute to the insemination of hundreds of sows through artificial insemination (AI), amplifying the biological and economic importance of semen quality parameters in intensive pig production systems (2). Among semen quality indicators, sperm motility and kinematic characteristics are considered critical predictors of fertilizing potential. These traits are commonly evaluated using computer-assisted sperm analysis (CASA) systems, which have been shown to correlate with pregnancy and farrowing outcomes in livestock species (3, 4). Even minor alterations in sperm motility profiles can have important implications for reproductive efficiency in commercial breeding systems.

Spermatozoa are sensitive to changes in their surrounding physicochemical environment, and various external contaminants introduced during semen collection or storage may negatively affect sperm function (5). Bacterial contamination of boar semen is a well-recognized factor influencing sperm functionality and storage capacity in artificial insemination systems (6). The presence of opportunistic bacteria within semen may negatively affect sperm motility, membrane integrity, and overall fertilizing potential (7). In addition, chemical compounds leaching from multilayer plastic semen bags have been shown to disrupt sperm function and reduce fertility outcomes in commercial pig farms (8, 9). These findings illustrate that spermatozoa respond quickly to environmental stressors and that the seminal microenvironment can be influenced by multiple, sometimes overlooked, sources of contamination.

Microplastics (MPs), typically defined as plastic particles <5 mm in size, are now recognized as widespread environmental pollutants detected in terrestrial, aquatic, and atmospheric systems (10). Exposure in humans and animals may occur through contaminated feed and water, airborne particles, or contact with synthetic materials used in agriculture and animal housing (11–13). Due to their small size, persistence, and capacity to adsorb chemical additives or environmental pollutants, MPs have raised concerns regarding potential biological and toxicological effects, including impacts on reproductive function in experimental animal models (14, 15).

Recent studies have begun to document MPs in reproductive tissues and fluids. MPs have been reported in human testicular tissue and semen (16, 17). MPs have similarly been identified in semen samples of other mammalian species, including dogs and bulls, where plastic particle adhesion to spermatozoa has been associated with reduced motility and lower fertilization rates under experimental conditions (18–20). In livestock production, additional MPs exposure sources may include housing materials, equipment components, water delivery systems, and environmental dust (13, 21). Farm-specific environmental conditions may therefore influence systemic MPs burden and potentially affect the presence of MPs in reproductive fluids (22). MPs can also adsorb endocrine disrupting chemicals, heavy metals, and other environmental pollutants, potentially increasing the bioavailability of biologically active compounds (23). Recent environmental microbiology studies have suggested that MPs may serve as substrates for microbial attachment and biofilm formation, potentially influencing bacterial persistence and antimicrobial susceptibility profiles (24, 25). Consequently, the co-occurrence of MPs and bacteria within biological matrices such as semen may represent an additional, yet insufficiently explored, factor of semen quality in livestock production systems.

Despite growing interest in the biological impacts of MPs, information on their occurrence and potential significance in the reproductive fluids of livestock species is still lacking. No studies to date have characterized the presence, polymer types, or size distribution of MPs in boar semen, nor explored whether MPs may be linked to functional semen characteristics. Given the significant role of boar semen in AI programs and the known sensitivity of spermatozoa to extracellular stressors, investigating MPs within the seminal microenvironment is relevant both scientifically and practically. The aim of this study was to characterize the presence and physicochemical properties of MPs in undiluted boar semen and to explore potential associations between MPs, CASA derived sperm motility parameters, bacterial load, and antimicrobial susceptibility profiles of bacterial isolates.

## 2 Materials and methods

The study was conducted at the Lithuanian University of Health Sciences (LSMU) in Kaunas, Lithuania, and the MPs analysis was performed at the Center for Physical Sciences and Technology (FTMC) in Vilnius, Lithuania. All procedures were carried out in accordance with the established ethical standards and received formal approval from the Lithuanian University of Health Sciences Bioethics Centre (Approval No.: 2024-BEC3-T-007).

### 2.1 Animals

Semen samples were collected from 12 clinically healthy mature boars aged 1–5 years. Animals were maintained at two independent pig breeding facilities, hereafter referred to as Farm A and Farm B. Farm A represented a commercial breeding herd comprising six boars from intensive production breeds (four Landrace and two Duroc). Farm B represented a genetic resource conservation herd comprising six boars from local native breeds (four Lithuanian White and two Lithuanian Indigenous pigs). The animals were housed and fed in accordance with national animal welfare regulations. Semen collection from these boars was performed routinely as part of standard reproductive management practices at both facilities. Ejaculates included in the present study were randomly selected from regularly collected semen samples. The study included ejaculates that satisfied the standard initial quality criteria applied in the collection of commercial boar semen, including normal appearance and the absence of visible contamination.

### 2.2 Semen collection and sample handling

A total of 24 sperm samples (*n* = 12 ejaculates, each collected in 2 separate samples) were collected. The sperm-rich fraction was obtained manually using the gloved-hand method. The operator wore nitrile gloves to ensure hygienic handling. The gloves did not come into direct contact with semen samples collected for MPs analysis, as ejaculate was obtained directly into collection containers. 20 ml of semen was collected into a glass container for MPs analysis, while 10 ml was collected separately into sterile plastic tubes for semen quality assessment and microbiological examination.

Samples collected in sterile plastic tubes were subdivided for semen quality assessment and bacteriological analysis. For semen quality evaluation, 1 ml of semen was transferred into sterile tubes using sterile pipette tips and transported to the Animal Reproduction Laboratory (LSMU) in a thermos maintained at 35 ± 2 °C within 60 min. Duplicate aliquots intended for microbiological analysis were transported to the Animal Microbiology Laboratory (LSMU) within 60 min in an insulated container with cooling elements. Samples collected in glass containers were transported to the Center for Physical Sciences and Technology (FTMC) for MPs analysis.

### 2.3 Microplastics analysis

Once arrived at the laboratory, samples were transferred from glass containers into 500 ml beakers. They were treated with hydrogen peroxide (H_2_O_2_), which was added dropwise using a pipette. During the initial treatment period, approximately 0.5–1 mL was added per hour, later increasing to 2–3 ml per hour. After each addition, the samples were stirred with a glass rod and heated in a drying oven at 40 °C. The treatment with H_2_O_2_ and heating was carried out for a period of 10–14 days, depending on sample quantity and complexity. The resistance of major synthetic polymers to oxidative digestion and the efficiency of biogenic organic matter removal have been evaluated in previous methodological studies (26). Later, the samples were filtered through paper filters and again rinsed with distilled water into clean beakers, after which 1–1.5 ml of concentrated hydrochloric acid (HCl) was added. The samples were then subjected to HCl treatment for 2–4 days. After this stage, the remaining material was transferred onto aluminum oxide filters. Finally, the aluminum oxide filters were dried in a drying oven at 60 °C for 10–20 min. The chemical composition and physical parameters of the MPs were determined using μ-FTIR LUMOS II (Bruker Optics GmbH, Ettlingen, Germany) FPA imaging mode. Polymer identification was carried out in the spectral range of 4,000–1,200 cm^−1^. The obtained data was compared with the instrument library data, and particles with a >70% match were identified as MPs.

#### 2.3.1 Environmental contamination control

To assess the potential for airborne MPs contamination during semen collection, environmental control samples were collected in parallel with the semen samples. A passive deposition approach, commonly used for monitoring airborne MPs in MPs analyses, was applied (27). Semen samples (*n* = 12 ejaculates) were collected across five distinct days of sampling, with one environmental control sample obtained on each sampling day. On each scheduled sampling day, an open glass Petri dish (90 mm) containing a paper filter was placed in the semen collection environment for passive airborne particle deposition. Prior to usage, all Petri dishes were rinsed with filtered distilled water. The Petri dishes were left open in the sampling area for a period of 3–8 h. The duration of exposure differed due to practical constraints present at the sampling sites. After this procedure, the Petri dishes were closed, sealed and were then transported along with the semen samples for MPs analysis. The results of the environmental control were used to qualitatively assess background airborne MPs contamination during sampling. Comparison of airborne MPs deposition rates per hour with MPs concentrations detected in semen samples did not reveal a proportional relationship between environmental MPs abundance and semen MPs levels. Consequently, airborne deposition during the brief semen exposure period was considered unlikely to substantially influence the measured MPs concentrations in semen. Correction was therefore not applied, as differences in exposure duration and deposition dynamics between environmental controls and semen samples which would introduce additional uncertainty into particle concentration estimates.

In addition, procedural laboratory blanks were included during MPs extraction and characterization. Ultrapure water blanks, which were filtered multiple times prior to use, did not contain detectable MPs. Passive environmental sedimentation controls collected during laboratory processing predominantly contained fibrous particles exceeding 2–3 mm in length, which were not detected in boar semen samples. Because both the quantity and particle profiles of background contamination differed from those observed in semen samples, quantitative correction or mathematical subtraction was considered inappropriate and potentially misleading.

### 2.4 Sperm quality assessment

#### 2.4.1 Sperm motility assessment

Upon arrival at the Animal Reproduction Laboratory, semen samples were allowed to equilibrate to laboratory conditions and were maintained at 35 ± 2 °C prior to dilution. Semen samples were diluted using a commercial extender Beltsville Thawing Solution (BTS) without antibiotic (Minitube, Tiefenbach, Germany) prewarmed to the same temperature to obtain a final sperm concentration of approximately 30–40 million spermatozoa/ml, to avoid temperature-induced motility alterations (28). Sperm motility was assessed using a CASA system (SCA^®^ version 6.5.0.91, Microptic S.L., Barcelona, Spain). Prior to CASA analysis, each sample was gently and consistently mixed by pipetting (3–5 times) to ensure homogeneous distribution of spermatozoa and MPs, and analysis was performed immediately thereafter to minimize potential sedimentation effects. A 10 μl aliquot of the diluted semen was carefully loaded onto the 10 μm depth Makler^®^ sperm counting chamber (Sefi Medical Instruments Ltd., Haifa, Israel), prewarmed to 37 ± 0.5 °C, by placing the droplet at the edge of the coverslip and allowing the sample to distribute evenly across the chamber. Care was taken to avoid bubble formation and overflow, and only fields showing visually homogeneous sperm distribution were selected for analysis. For each sample, three technical replicates were analyzed. In each replicate, sperm motility was evaluated in at least four non-overlapping fields, resulting in a minimum of 12 fields analyzed per sample. This approach ensured the analysis of at least 1,000 spermatozoa per sample, in accordance with recommended CASA practices for reliable motility assessment (29). The following motility and kinematic parameters were evaluated: total motility, progressive motility, velocity categories classified as rapid velocity, medium velocity, and slow velocity spermatozoa, as well as curvilinear velocity (VCL), straight-line velocity (VSL), average path velocity (VAP), linearity (LIN), straightness (STR), wobble coefficient (WOB), amplitude of lateral head displacement (ALH), and beat cross frequency (BCF). The CASA system settings were configured according to the manufacturer's recommendations for boar semen analysis. Image acquisition was performed at 30 frames per second, in accordance with standard CASA settings for boar semen analysis (4). Sperm detection and tracking parameters were kept constant throughout the study. All measurements were performed by the same trained operator under standardized conditions to ensure consistency and repeatability.

#### 2.4.2 Sperm morphology assessment

After arrival at the Animal Reproduction Laboratory, boar semen samples were gently homogenized by pipetting. A 10 μl aliquot of each sample was placed onto a clean glass slide, smeared, air-dried, and subsequently stained using the SpermBlue staining kit (Microptic SL, Barcelona, Spain) according to the manufacturer's instructions and established protocols. Morphological evaluation was performed using a light microscope (Eclipse 50i, Nikon, Japan) equipped with an oil immersion objective at 1,000 × magnification. For each sample, a total of 200 spermatozoa were examined in a systematic manner to ensure representative assessment of sperm morphological variability. Spermatozoa were classified as morphologically normal or abnormal. Morphological abnormalities were categorized into three main groups: head defects, midpiece defects, and tail defects. Head defects included detached pathological heads, pyriform head, narrow-based head, abnormal head contour, undeveloped spermatozoa, narrow head, macrocephaly, microcephaly (normal shape), short and broad head, and paracentric midpiece implantation. Midpiece defects comprised proximal and distal cytoplasmic droplets as well as other midpiece abnormalities. Tail defects included coiled tail, tail coiled around the head, double-bent tail, and detached tail (tailless spermatozoa). All observed morphological abnormalities were recorded in standardized data sheets. The proportions of each abnormality category and the total percentage of morphologically abnormal spermatozoa were calculated.

### 2.5 Bacterial analysis and antimicrobial susceptibility testing

For bacterial analysis, semen samples were serially diluted in sterile physiological saline. Aliquots (10 μl) of each dilution were spread onto 90 mm blood agar plates in triplicate and incubated aerobically at 35 ± 2 °C for 24 h. After incubation, colony-forming units (CFU) were counted, and results were expressed as CFU/ml of semen. Colonies differing in morphology were selected for subculturing to obtain pure isolates. Bacterial identification was performed using matrix-assisted laser desorption/ionization-time of flight mass spectrometry (MALDI Biotyper^®^ Sirius; Bruker Daltonics, Bremen, Germany), following the manufacturer's standard operating procedures (30). Bacterial isolates considered potentially relevant to semen quality based on their abundance and known opportunistic potential in boar semen were further subjected to antimicrobial susceptibility testing using minimum inhibitory concentration (MIC) gradient strips (MTS™, Liofilchem™, Roseto degli Abruzzi, Italy). The antibiotics tested included penicillin, gentamicin, spectinomycin, streptomycin, and amoxicillin, representing antimicrobial agents commonly used in boar semen extenders in accordance with European regulatory requirements (Commission Delegated Regulation (EU) 2020/686, as amended by Regulation (EU) 2023/647) (31, 32). MIC testing was performed according to standardized procedures (33). Briefly, pure bacterial cultures were suspended in sterile saline and adjusted to a turbidity equivalent to 0.5 McFarland standard. The bacterial suspension was evenly spread onto 150 mm Mueller-Hinton agar plates, MIC gradient strips were applied, and plates were incubated at 35 ± 1 °C for 18–24 h. MIC values were determined visually at the point of complete growth inhibition, in accordance with the manufacturer's instructions.

### 2.6 Statistical analysis

Data were organized in Microsoft Excel (Microsoft Office Standard 2019) and statistical analyses were performed using IBM SPSS Statistics (version 30.0, IBM Corp., Armonk, NY, USA). Due to the limited sample size, nonparametric statistical tests were applied throughout the study. Descriptive statistics are presented as median (interquartile range Q_1_, Q_3_) and minimum–maximum values. Differences in MPs concentrations between farms were assessed using the Mann–Whitney *U*-test. In addition to *p*-values, effect sizes (r) for Mann–Whitney *U*-test were calculated. Boar semen samples were stratified into two groups based on MPs concentration to compare sperm morphology parameters: low MPs contamination group (<10 MPs/ml; *n* = 6) and the high MPs contamination group (≥10 MPs/ml; *n* = 6). Associations between MPs characteristics (polymer-specific concentrations and particle size fractions) and semen quality parameters / bacterial MIC results were evaluated using Spearman's rank correlation. Correlation coefficients (ρ) and corresponding *p*-values are reported, with emphasis on the direction and magnitude of associations. Confidence intervals (95% CI) for Spearman's rho were calculated and are provided in the Supplementary material. To control for multiple testing in correlation analyses, *p*-values were adjusted using the Benjamini–Hochberg false discovery rate (FDR) procedure. FDR correction was applied separately for each test family (e.g., CASA motility parameters across polymer-specific correlations). Raw and FDR-adjusted *p*-values are provided in the Supplementary material. For correlation analyses, statistical significance was defined as *p*(FDR) <0.05.

## 3 Results

### 3.1 Occurrence and concentration of microplastics in boar semen

**Figure 1:**
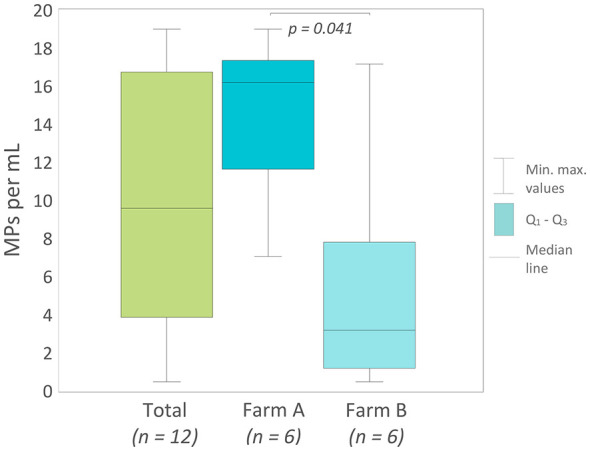
Distribution of total microplastics (MPs) concentrations in undiluted boar semen and comparison between Farm A and Farm B. Boxplots display the median (central line) and interquartile range (Q_1_, Q_3_), with whiskers representing minimum and maximum values (MPs/ml). A statistically significant difference in MPs concentrations between Farm A and Farm B was identified (Mann–Whitney *U*-test, *p* = 0.041).

The presence of MPs was detected in all undiluted boar semen samples (*n* = 12). The total concentration of MPs ranged from 0.48 to 19.00 MPs/ml, with a median value of 9.58 MPs/ml (Q_1_, Q_3_: 3.86, 16.74) (Figure 1). The concentration values exhibited variability among the samples.

MPs concentrations differed significantly between the two farm groups (Figure 1). The median (min–max) MPs concentration in the semen samples from Farm A (*n* = 6) was 16.2 (7.06–19.00) MPs/mL, whereas the median of the samples from Farm B (*n* = 6) was 3.2 (0.48–17.16) MPs/ml. Notably, a wider range of MPs concentrations was observed in samples from Farm B. A statistically significant difference between the groups was identified using the Mann–Whitney *U*-test (*U* = 5.0; *p* = 0.041), with a large effect size (*r* = 0.60).

MPs were also detected in environmental control samples collected during sampling. Their abundance and composition varied between sampling days (Supplementary Table S1a). Comparison of airborne MPs deposition rates with MPs concentrations detected in semen samples did not indicate a direct proportional relationship between environmental contamination levels and semen MPs concentrations (Supplementary Table S1b).

### 3.2 Polymer composition of detected microplastics

**Figure 2:**
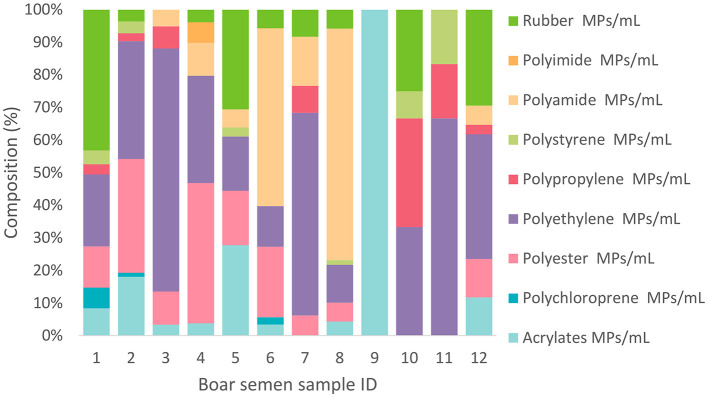
The figure presents the percentage distribution of microplastics (MPs) chemical composition, defined as the proportion of identified polymer types among MPs per sample, as determined by Fourier-transform infrared (FTIR) analysis.

A total of nine distinct polymer types were identified across the entire dataset by Fourier-transform infrared (FTIR) spectroscopy (Figure 2). Polyethylene was the most frequently detected polymer (11/12 samples), followed by polyester, rubber, and acrylates (each detected in 9/12 samples). Other polymers, such as polystyrene (6/12) and polychloroprene (3/12), were detected occasionally, while polyimide was identified in a single sample (1/12). The number of polymer categories present in each sample varied between one and seven.

### 3.3 Size distribution of microplastics

**Figure 3:**
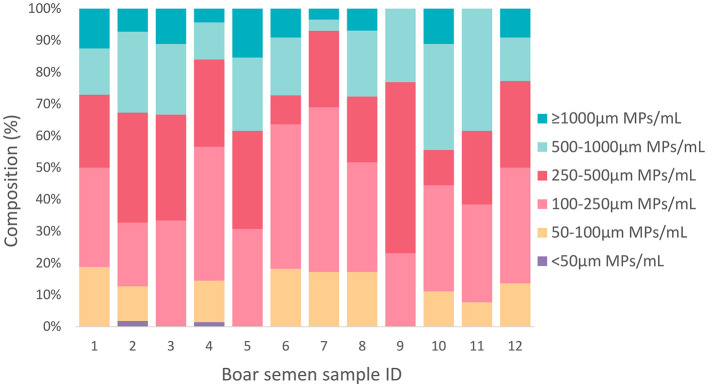
The diagram illustrates the distribution of microplastic particles by size categories, expressed as a percentage of the total in sample. The range of sizes considered in this study was less than 50 μm, from 50 μm to 100 μm, from 100 μm to 250 μm, from 250 μm to 500 μm, from 500 μm to 1,000 μm, and larger than 1,000 μm. Particles that matched the lower limit of each range were assigned to that category.

MPs were most frequently detected within the 100–500 μm size range. Particles <50 μm and ≥1,000 μm were detected least frequently (Figure 3).

### 3.4 Associations between microplastics and semen motility parameters

**Figure 4:**
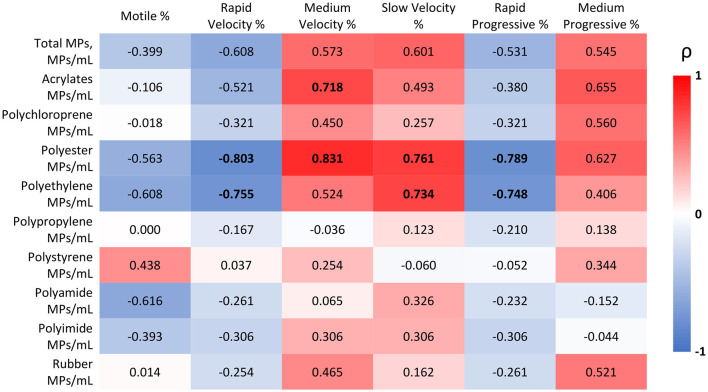
The correlation heatmap presents a summary of Spearman's rank correlation coefficients (ρ) between microplastics (MPs) concentrations (MPs/ml) and boar sperm motility parameters assessed by computer assisted sperm analysis (CASA). The intensity of coloration functions as a quantitative metric for the strength and direction of correlations, with blue indicating negative and red signifying positive associations. Spearman's rho correlation coefficients (ρ) are displayed within each cell. Bold values indicate correlations that remained statistically significant after Benjamini–Hochberg false discovery rate (FDR) correction (*p*(FDR) <0.05). Confidence intervals and corresponding *p*-values are provided in the Supplementary Table S2.

A non-parametric method was applied to quantify the direction (sign of ρ) and magnitude (absolute value of ρ) of associations between variables without implying causal relationships. Spearman's rank correlation analysis revealed several moderate to strong associations between MPs concentrations and boar sperm motility parameters evaluated using CASA (Figure 4).

Following adjustment for multiple comparisons using the Benjamini–Hochberg false discovery rate, only the most robust and consistent associations remained statistically significant. Higher concentrations of polyester and polyethylene MPs were significantly and negatively associated with the proportion of spermatozoa exhibiting rapid velocity and rapid progressive motility, while positive associations with slow velocity fractions also persisted after FDR correction. Acrylates MPs concentrations also showed a statistically significant positive association with medium velocity fractions.

Associations involving total MPs concentrations and other polymer types (including polychloroprene, polypropylene, polystyrene, polyamide, polyimide, and rubber) were weaker and did not show consistent patterns across motility parameters following FDR adjustment.

Confidence intervals for Spearman's rho and *p*-values adjusted for multiple comparisons are provided in the Supplementary Table S2. Confidence intervals were generally wide, reflecting uncertainty related to the limited sample size and indicating that effect size estimates should be interpreted with caution.

### 3.5 Associations between microplastic size groups and semen motility parameters

**Figure 5:**
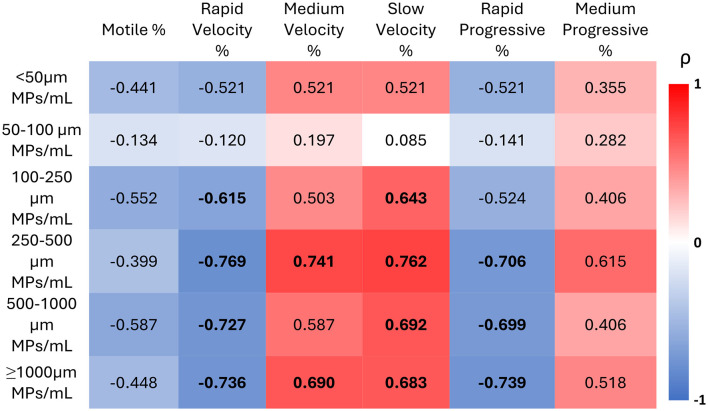
The correlation heatmap presents a summary of Spearman's rank correlation coefficients (ρ) between microplastic particles (MPs) size classes concentrations (MPs/mL) and boar sperm motility parameters measured by computer-assisted sperm analysis (CASA). Correlations are shown for six particle size fractions (<50 μm, 50–100 μm, 100–250 μm, 250–500 μm, 500–1,000 μm, and ≥1,000 μm, particles that matched the lower limit of each range were assigned to that category). Color intensity represents the direction and magnitude of correlations, with blue indicating negative and red signifying positive values. Spearman's rho correlation coefficients (ρ) are displayed within each cell. Bold values indicate correlations that remained statistically significant after Benjamini–Hochberg false discovery rate (FDR) correction (*p*(FDR) <0.05). Confidence intervals and corresponding *p*-values are provided in the Supplementary Table S3.

Spearman's rank correlation analysis was performed to evaluate the relationships between MPs particle size categories concentration (MPs/ml) and boar sperm motility parameters evaluated using CASA (Figure 5). Following adjustment for multiple comparisons using the Benjamini–Hochberg FDR, several MPs size fractions ≥100 μm demonstrated statistically significant associations with sperm motility parameters. Across these size classes, negative associations were consistently observed with rapid velocity and rapid progressive motility, while positive associations persisted with medium and slow velocity fractions.

The strongest and most consistent associations were observed for the 250–500 μm size fraction. This fraction showed significant negative correlations with rapid velocity (ρ = −0.769, *p*(FDR) = 0.021) and rapid progressive motility (ρ = −0.706, *p*(FDR) = 0.031), as well as significant positive correlations with medium velocity (ρ = 0.741, *p*(FDR) = 0.035) and slow velocity (ρ = 0.762, *p*(FDR) = 0.024). Similar directional patterns were observed for the 100–250 μm, 500–1,000 μm, and ≥1,000 μm size fractions, with selected velocity parameters remaining statistically significant after FDR correction. In contrast, the smallest particle size fractions (<50 μm and 50–100 μm) exhibited weaker and less consistent associations with motility parameters, and no correlations remained statistically significant after FDR adjustment. Confidence intervals for Spearman's rho and *p*-values adjusted for multiple comparisons are provided in the Supplementary Table S3. Confidence intervals were generally wide, reflecting uncertainty related to the limited sample size and indicating that effect size estimates should be interpreted with caution.

### 3.6 Associations between microplastics and spermatozoa morphology

No statistically significant associations were observed between MPs concentrations and sperm morphological abnormalities (head defects, midpiece defects, tail defects, or total pathological abnormalities percent) using Spearman's rank correlation analysis (all *p* > 0.05; Supplementary Table S4). Furthermore, comparison between the low MPs contamination group (<10 MPs/ml; *n* = 6) and the high MPs contamination group (≥10 MPs/ml; *n* = 6) revealed no significant differences in any sperm morphology defects categories (Mann–Whitney *U*-test, all *p* > 0.05).

### 3.7 Associations between microplastics, bacterial load, and antimicrobial susceptibility

Bacterial CFU in boar semen, had a median (min–max) of 25,966 (1,300–178,500) CFU/ml. No statistically significant correlations were detected between bacterial contamination and MPs concentrations or MPs size fractions. The most frequently detected bacterial species in the semen samples were *Escherichia coli*, identified in 75% of samples (9 of 12), *Staphylococcus equorum*, detected in 58.3% of samples (7 of 12), and *Pseudomonas aeruginosa*, detected in 50% of samples (6 of 12). These isolates were then tested for antimicrobial susceptibility using MIC gradient strips, and the resulting MIC values were included in correlation analyses with MPs variables. Following adjustment for multiple comparisons using the Benjamini–Hochberg false discovery rate, most associations between MPs concentrations and MIC values did not remain statistically significant (Supplementary Table S5).

For *E. coli* isolates (*n* = 9), several correlations were observed at the unadjusted *p*-value level. However, after FDR correction, no statistically significant associations remained, with the exception of a strong negative correlation between amoxicillin MIC values and polystyrene MPs concentrations (ρ = −0.915, *p*(FDR) = 0.006). For *P. aeruginosa* isolates (*n* = 6), none of the observed associations between MPs concentrations and MIC values remained statistically significant after FDR correction. In contrast, for *S. equorum* isolates (*n* = 7), statistically significant negative associations persisted after FDR correction between spectinomycin MIC values and both polypropylene (ρ = −0.933, *p*(FDR) = 0.019) and polystyrene MPs concentrations (ρ = −0.907, *p*(FDR) = 0.022). No statistically significant correlations were observed between MPs variables and gentamicin, streptomycin, amoxicillin, or penicillin MIC values for *S. equorum*.

## 4 Discussion

MPs were detected in every analyzed boar semen sample, indicating that particulate contaminants can enter the boar reproductive tract, even under standard farm conditions. This represents a previously unrecognized component of the boar seminal microenvironment. The occurrence of MPs in reproductive fluids has previously been reported in other mammalian species, including bull epididymal semen and dog semen samples, where MPs were also detected in all analyzed samples (19, 20). Reported concentrations reached approximately 72.5 MPs/ml in bovine epididymal sperm and 35.4 MPs/ml in canine seminal plasma. These values were higher than those observed in our boar samples. This difference may reflect species specific exposure pathways or variation in MPs accumulation within reproductive fluids. Research involving human studies detected MPs in all participants (113 male) semen and urine samples tested, with participants typically exposed to multiple types simultaneously (34). In our research concentrations varied substantially among individual boars and between two distinct breeding facilities. The particularly wide range observed in Farm B suggests pronounced heterogeneity in exposure levels within this farm. Such dispersion likely arises from the combined influence of individual-specific factors (e.g., differences in feeding behavior, housing location within the facility, or micro-environmental conditions) and uneven environmental MPs distribution. Although the sample size was limited, the observed difference between farms was associated with a large effect size, suggesting that variation in MPs concentrations may be depended on environmental factors. The observed variability among individual samples and the significant difference between breeding facilities together suggest heterogeneous exposure patterns potentially influenced by specific environmental or management factors associated with farms. Differences in housing systems, feed sources, environmental hygiene, and surrounding pollution levels could therefore potentially contribute to variability in systemic MPs exposure in livestock production systems (13).

Environmental control samples confirmed the presence of airborne MPs in the sampling environment. However, comparison of airborne MPs deposition rates with MPs concentrations detected in semen samples did not reveal a consistent or proportional relationship. Higher airborne MPs deposition was not associated with increased MPs concentrations in semen. Considering the brief exposure of semen to ambient air during collection and prompt sample covering, short-term airborne contamination was therefore considered unlikely to substantially influence the MPs concentrations measured in semen. These findings support the interpretation that at least part of the MPs detected in semen originates from systemic exposure pathways, such as ingestion or inhalation of MPs present in the farm environment, followed by distribution to reproductive fluids, rather than sampling-related contamination. Furthermore, laboratory procedural blanks confirmed the absence of MPs in ultrapure water controls and identified only large fibrous particles (>2–3 mm) in environmental sedimentation samples that were not observed in semen. The lack of overlap between background contamination profiles and semen MPs composition supports the interpretation that mathematical correction based on environmental controls would be inappropriate.

In addition to detecting MPs in all samples, the present study characterized the polymer composition of the MPs particles. Polyethylene was the most frequently identified polymer, followed by polyester, rubber, and acrylates. Polymer composition reported in reproductive samples varies across species and studies. In bovine and canine semen, polystyrene and polypropylene particles have been frequently detected (20). Studies in human semen samples have also identified polyethylene as one of the most abundant polymers in semen samples as in the current study of boar semen (16, 35). However, other research in human reproduction contamination of MPs showed variability of chemical composition of particles (17). The predominance of polyethylene aligns with the widespread use of this polymer in agriculture and packaging, where it is one of the most common polymers found in environmental and biological samples (36, 37). Furthermore, recent analyses indicate that polyethylene, polypropylene, and polystyrene frequently appear as the most abundant types among polymers detected in human and environmental matrices. This observation reflects their production volumes and usage patterns (38).

The partial overlap in polymer types across species may be indicative of the environmental distribution of these materials, rather than species-specific accumulation patterns (38). The observed heterogeneity in polymer composition among individual samples may reflect exposure to multiple and variable sources within livestock production systems (12). The presence of acrylates and rubber-associated particles in several samples may be linked to materials commonly used in farm environments, including flooring materials, equipment components, or vehicle-derived particles present in farm surroundings (39–41). Polyethylene and polypropylene are extensively used in feed packaging materials, plastic storage containers, and water distribution systems, making them likely contributors to oral exposure pathways (42). Polyester fibers are frequently associated with synthetic textiles, such as clothing or protective gear, and are also commonly reported as airborne fibers in agricultural and indoor environments (43, 44). Additionally, airborne MPs originating from dust, ventilation systems, or external environmental contamination may further contribute to the diversity of polymer types detected (45). Although these potential sources were not directly investigated in the present study, the observed diversity of polymers suggests that animals are likely exposed to complex and heterogeneous mixtures of MPs from multiple environmental pathways. This contrasts with the simplified exposure scenarios commonly applied in experimental studies, where single polymer types - most frequently polystyrene microparticles - are used to assess biological effects (46–50). Further investigations incorporating environmental sampling and source attribution approaches would be necessary to better characterize the origin and relative contribution of specific polymer sources in livestock production systems.

In the present study, exploratory correlation analyses revealed multiple moderate to strong associations between MPs concentrations and sperm motility parameters assessed by CASA. At the unadjusted level, higher total MPs concentrations tended to be associated with a lower proportion of spermatozoa exhibiting rapid velocity and higher proportions of slow velocity categories. However, these associations did not consistently remain significant after correction for multiple comparisons. Polymer-specific analyses indicated that this pattern was particularly evident for polyethylene and polyester particles. Elevated concentrations of these polymers were negatively correlated with rapid velocity and rapid progressive motility parameters, while positive correlations were observed with higher percentage of slow velocity spermatozoa.

Experimental studies in animal models and in vitro human studies have also reported associations between MPs exposure and reduced sperm motility (16, 47, 51). The observed associations between higher MPs levels and altered sperm kinematic profiles may be consistent with biological mechanisms previously described in experimental studies (14, 51, 52). Whilst the present study was not designed to evaluate molecular pathways, the available evidence suggests that exposure to MPs has been associated with oxidative stress, inflammatory responses, and cellular dysfunction within the male reproductive system (14). Studies in swine testicular cells have demonstrated that polystyrene MPs trigger oxidative signaling and cell death pathways, offering a plausible mechanism for MPs related sperm dysfunction (48). Polystyrene particles induce apoptosis and necroptosis via Reactive oxygen species (ROS)/ mitogen-activated protein kinase/ Hypoxia-inducible factor (ROS/MAPK/HIF1α) signaling cascades (48). In a recent human cohort study analyzing nanoplastics (NPs) in seminal plasma, higher polyvinyl chloride concentrations were associated with reduced sperm motility, while elevated polyethylene and polyvinyl chloride levels in follicular fluid were linked to decreased fertilization rates during in vitro fertilization (52). In addition, in vitro studies on human sperm exposed to polystyrene MPs have reported reduced motility, increased ROS production, sperm DNA fragmentation, and sperm agglutination, suggesting both oxidative and physical interaction mechanisms (51).

Experimental in vitro evidence further supports the potential role of mitochondrial dysfunction in MPs induced reproductive effects. Disruptions in mitochondrial function are well established contributors to impaired sperm motility and altered kinematic parameters, as ATP production and membrane potential are critical for proper movement (53). Exposure of mouse spermatocyte GC-2 cells line to 5 μm polystyrene MPs has been shown to decrease ATP content and significantly reduce mitochondrial membrane potential, accompanied by oxidative stress damage to mitochondrial structure and activation of the PTEN-induced putative kinase 1 (PINK1) /Parkin-mediated mitophagy pathway (54). Accordingly, experimental studies have demonstrated that exposure to polystyrene MPs can impair sperm function and fertilization capacity in mammalian experimental models, potentially mediated by increased oxidative stress and mitochondrial dysfunction (19, 47). Such effects have been proposed to occur through direct physical interactions between particles and spermatozoa, as polystyrene MPs have been shown to adhere to sperm cells, increase oxidative stress and reduce motility and fertilization capacity in mammalian models (19). In human observational studies, exposure to specific polymer types such as polyethylene terephthalate has also been associated with reduced progressive sperm motility (16). Plastic compounds have also been reported to affect boar sperm quality under commercial conditions. For example, leaching of bisphenol A diglycidyl ether (BADGE) from multilayer semen bags was associated with impaired motility, altered kinematic parameters, reduced mitochondrial activity, and decreased farrowing rates in sow herds (9). Although the biological behavior of particulate MPs differs from that of dissolved plastic additives, these findings indicate that plastic-related contaminants, whether in the form of leached chemical additives or particulate MPs, may represent a previously underrecognized environmental pollution factor potentially associated with variation in the seminal microenvironment and sperm motility characteristics.

Furthermore, particle size distribution may be biologically relevant, as the strongest associations with altered sperm motility were observed for intermediate size fractions, particularly those measuring 250–500 μm. Although smaller MPs and NPs can penetrate cellular barriers (12, 14, 47), a notable finding of the present study was that the strongest associations between MPs and sperm motility parameters involved relatively large particle size fractions (250–500 μm). Given their size, these particles are unlikely to penetrate spermatozoa membranes or exert direct intracellular effects. Instead, the observed associations may be best explained by indirect physical mechanisms, such as mechanical interference with sperm progression, localized particle–sperm contact, or changes in the microenvironment of the seminal plasma affecting sperm trajectories. Larger particles are also more prone to sedimentation and heterogeneous distribution within biological fluids, which could facilitate localized interactions with motile spermatozoa despite standardized sample handling. Alternatively, the presence of larger MPs may reflect broader environmental MPs exposure, acting as indicators of associated plastic-derived contaminants not captured by the applied analytical approach. However, distinguishing between these potential mechanisms was beyond the scope of the present study and warrants further investigation.

Despite the consistent detection of MPs in all semen samples, no statistically significant associations were observed between MPs concentrations and sperm morphological abnormalities in the present study. Neither total MPs burden, polymer-specific MPs, nor particle size fractions were correlated with the percentage of head, midpiece, tail defects, or total pathological spermatozoa. These findings suggest that, at the exposure levels observed in this study, the presence of MPs was not associated with detectable structural alterations in spermatozoa. The absence of detectable associations with sperm morphology may reflect differences in sensitivity among sperm quality parameters. Sperm motility and kinematic parameters are considered to be early and highly responsive indicators of subcellular dysfunction, whereas morphological abnormalities usually indicate more advanced or irreversible structural damage that occurs during spermatogenesis. Therefore, the effects of MPs may initially be present as functional impairments, such as altered motility, without producing measurable morphological defects. This interpretation is supported by experimental studies demonstrating that exposure to MPs can disrupt mitochondrial function, oxidative balance and sperm motion without immediately altering sperm morphology (47). Furthermore, while morphological assessment based on light microscopy is relevant for detecting gross structural abnormalities, it may lack sensitivity in identifying subtle ultrastructural or molecular alterations that could precede visible morphological damage.

Beyond MPs potential effects on sperm functionality, MPs may also influence microbial dynamics within biological fluids. In the present study, the most frequently detected bacterial species were *E. coli, S. equorum*, and *P. aeruginosa*, which are commonly reported among environmental or opportunistic bacteria associated with boar semen contamination during collection and handling (6, 55). MPs have been shown to serve as surfaces for microbial attachment and biofilm formation in environmental systems, a phenomenon commonly referred to as the “plastisphere” (56, 57). This led us to explore whether MPs might also interact with bacteria present in boar semen.

No statistically significant associations were identified between overall bacterial contamination levels, expressed as CFU/mL, and MPs concentrations or size fractions. These findings indicate that MPs were not associated with overall bacterial abundance under the conditions of the present study. The absence of correlations between overall bacterial load and MPs may reflect the multifactorial nature of microbial contamination in boar semen, which is influenced by environmental hygiene and host microbiota (28, 58). Several associations between MPs concentrations and antimicrobial susceptibility profiles were observed at the unadjusted *p*-value level. However, most of these relationships did not remain statistically significant after correction for multiple comparisons, highlighting their exploratory nature.

For *Escherichia coli* isolates, a positive association between spectinomycin MIC values and acrylate MPs concentrations were observed prior to FDR correction. Although this relationship did not remain statistically significant after adjustment, its direction is consistent with previous environmental and *in vitro* studies suggesting that certain MPs polymers may facilitate increased antimicrobial tolerance through biofilm formation or altered antibiotic bioavailability. Experimental studies have demonstrated that MPs can enhance biofilm-associated antimicrobial tolerance in *E. coli*, which could potentially contribute to increased MIC values under specific conditions (59). Interactions between bacteria and MPs have also been shown to promote horizontal gene transfer and alter bacterial stress responses in environmental systems (60, 61). However, as most evidence for these mechanisms originates from aquatic or soil environments (24, 25), the present findings should be interpreted cautiously and cannot be considered confirmatory.

In contrast, robust associations that remained statistically significant after FDR correction were identified for *Staphylococcus equorum*. Higher concentrations of polypropylene and polystyrene MPs were consistently associated with lower spectinomycin MIC values. These results suggest complex and potentially species-specific interactions between MPs and bacterial susceptibility patterns. While many studies have reported that MPs may promote antimicrobial resistance (24, 59, 60), the present results indicate that MPs may also be associated with increased susceptibility in certain bacteria species in boar semen. Such contrasting effects may reflect physicochemical interactions between MPs and antibiotics, including adsorption processes, as well as shifts in bacterial community composition or physiological states (62, 63). Further studies are required to clarify these mechanisms and to determine their relevance in biological fluids such as semen.

Although the median MPs concentration detected in boar semen in the present study (9.58 MPs/ml) cannot be directly interpreted in terms of clinical reproductive outcomes, such as fertilization success or litter size, these concentrations fall within a range at which functional associations with altered sperm motility parameters were observed. At present, no reference thresholds exist to define biologically safe or harmful MPs concentrations in semen, either for livestock species. Importantly, sperm motility and kinematic parameters are among the most sensitive and functionally relevant indicators of fertilizing capacity in boar artificial insemination programs (3, 4). Alterations in progressive motility, velocity distribution, and kinematic profiles have been consistently associated with reduced fertilization efficiency and impaired reproductive performance (64). In this context, the observed associations between the MPs in semen and sperm motility parameters suggest that exposure to MPs at under real-world exposure conditions may be functionally relevant, even in the absence of detectable effects on sperm morphology. The present cross-sectional study did not include fertility endpoint data, such as pregnancy rates or litter size, and therefore cannot establish whether the detected motility alterations result in measurable reductions in reproductive success. Future studies integrating semen MPs characterization with artificial insemination outcomes under commercial conditions will be essential to determine the clinical significance of MPs contamination in boar semen reservoirs.

The findings of the present study should be interpreted considering several limitations. First, the relatively small sample size (*n* = 12) limits statistical power and increases uncertainty around effect estimates. Although confidence intervals were provided and *p*-values were adjusted for multiple testing, the observed associations should be considered exploratory. Second, the cross-sectional design restricts causal inference, as detected relationships between MPs characteristics and sperm motility or antimicrobial susceptibility profiles represent correlations rather than temporal or causal effects. Third, semen samples were collected at a single time point, and environmental MPs sources were not systematically characterized. Additionally, the physicochemical behavior of MPs in semen extender, including potential sedimentation or agglomeration, was not directly assessed and may influence local particle distribution during analysis. Therefore, temporal variability in exposure and the relative contribution of specific sources could not be assessed. Finally, a substantial proportion of detected MPs consisted of relatively large particle size fractions whose biological relevance and indirect mechanisms of action remain insufficiently understood. Future studies incorporating longitudinal designs, environmental source tracking, and reproductive performance endpoints will be essential to clarify the biological and clinical significance of MPs contamination in boar semen.

## 5 Conclusion

This study confirms the presence of MPs in undiluted boar semen collected under routine farm conditions. Exploratory associations were also identified between MPs characteristics and CASA sperm motility parameters, as well as preliminary links with antimicrobial susceptibility patterns of most frequently isolated bacterial species. Together, these observations indicate that particulate contaminants can be part of the seminal microenvironment and may influence both sperm function and bacteria–antibiotic interactions in boar semen used for artificial insemination. The findings highlight an environmental factor that has received little attention in livestock reproduction. Further work will be needed to clarify the mechanisms involved and to determine how widespread and biologically relevant these contaminants are in practical pig breeding settings.

## Data Availability

The raw data supporting the conclusions of this article will be made available by the authors, without undue reservation.
