# Supplementary material for: Microplastics in the seminal microenvironment of boar semen: associations with sperm motility and antimicrobial susceptibility
Source: Front Vet Sci. 2026 May 26;13:1847076. doi: 10.3389/fvets.2026.1847076 (PMC13271002; doi:10.3389/fvets.2026.1847076)
Supplement: Supplementary file 1 [file Table_1.docx]

Supplementary Material

Table S1a. Overview of microplastic (MPs) particles detected in environmental control samples collected during semen sampling, including exposure duration, total particle counts, polymer composition, and size distribution categories (<50 µm, 50–100 µm, 100–250 µm, 250–500 µm, 500–1000 µm, and ≥1000 µm, particles that matched the lower limit of each range were assigned to that category).

| Sampling site (farm A/B) | Exposure duration (h) | Total MPs (particle count) | Chemical composition | | | | | | | | | | | Size categories | | | | | |
| --- | --- | --- | --- | --- | --- | --- | --- | --- | --- | --- | --- | --- | --- | --- | --- | --- | --- | --- | --- |
|  |  |  | Acrylates (%) | Polychloroprene (%) | Polyester (%) | Polyethylene (%) | Polypropylene (%) | Polystyrene (%) | Polyisoprene (%) | Polyamide (%) | Polyimide (%) | Polyvinylchloride (%) | Rubber (%) | <50µm (%) | 50-100µm (%) | 100-250µm (%) | 250-500µm (%) | 500-1000µm (%) | ≥1000µm (%) |
| A | **8** | **58** | 6.9 | 10.3 | 13.8 | 24.1 | 3.5 | ND | 3.5 | ND | ND | ND | 37.9 | ND | ND | 28.6 | 25.7 | 25.7 | 17.1 |
| A | **7** | **64** | 9.4 | ND | 6.3 | 12.5 | ND | ND | ND | 3.1 | ND | ND | 68.8 | 2.9 | 14.7 | 17.7 | 20.6 | 26.5 | 17.7 |
| A | **8** | **80** | 10.0 | ND | 25.0 | 35.0 | 5.0 | 10.0 | ND | ND | 5.0 | 5.0 | 5.0 | ND | 30.8 | 38.5 | 15.4 | 15.4 | ND |
| B | **3** | **60** | 6.7 | ND | ND | 80.0 | ND | 6.7 | ND | ND | ND | ND | 6.7 | ND | 6.3 | 37.5 | 18.8 | 31.3 | 6.3 |
| B | **3** | **144** | ND | ND | 2.8 | 55.6 | 5.6 | ND | ND | 22.2 | ND | ND | 13.9 | 10.0 | 20.0 | 10.0 | 30.0 | 30.0 | ND |

ND indicates that no particles of the respective polymer type or size category were detected in the sample.

Table S1b. Airborne microplastic (MPs) deposition rates measured in environmental control samples and corresponding MPs concentrations detected in undiluted boar semen samples.

| Sampling day | Airborne MPs deposition rate (particles/hour) | MPs concentration in boar semen (MPs/mL) |
| --- | --- | --- |
|  |  |  |
| 1 | 7.25 | 19.00 |
| 1 | 7.25 | 16.60 |
| 2 | 9.14 | 10.26 |
| 2 | 9.14 | 15.80 |
| 3 | 10.00 | 7.06 |
| 3 | 10.00 | 17.60 |
| 4 | 20.00 | 17.16 |
| 4 | 20.00 | 8.90 |
| 4 | 20.00 | 1.85 |
| 5 | 48.00 | 0.96 |
| 5 | 48.00 | 0.48 |
| 5 | 48.00 | 4.53 |

Note: Multiple semen samples were collected on the same sampling day, therefore, identical airborne MPs deposition rates correspond to different semen samples collected under the same environmental conditions. Values are reported to illustrate the lack of a direct proportional relationship between short‑term airborne MPs deposition and MPs concentrations detected in semen samples.
