# Supplementary material for: Microplastics in the seminal microenvironment of boar semen: associations with sperm motility and antimicrobial susceptibility
Source: Front Vet Sci. 2026 May 26;13:1847076. doi: 10.3389/fvets.2026.1847076 (PMC13271002; doi:10.3389/fvets.2026.1847076)
Supplement: Supplementary file 2 [file Table_2.docx]

Supplementary Material

Table S2. Spearman’s rank correlation coefficients (ρ), two-tailed significance levels (p-values), Benjamini–Hochberg false discovery rate (FDR)‑adjusted p‑values, and 95% confidence intervals (Bonett and Wright method) for correlations between microplastic (MPs) concentrations (total and polymer-specific particle counts, MPs/mL) and sperm motility parameters measured by computer-assisted sperm analysis (CASA) in boar semen samples (n = 12). Statistical significance after FDR correction was defined as p (FDR) < 0.05.

|  | Spearman's rho ρ | Significance (2-tailed)  p-value | p-value (FDR) | Significant after FDR (Yes/No) | 95% Confidence Intervals (2-tailed) | | |
| --- | --- | --- | --- | --- | --- | --- | --- |
|  |  |  |  |  | **Lower** | **Upper** | |
| Motile % - Total MPs, MPs/mL | -0.399 | 0.199 | 0.399 | No | -0.801 | 0.251 |  |
| Motile % - Acrylates, MPs/mL | -0.106 | 0.744 | 1.000 | No | -0.642 | 0.500 |  |
| Motile % - Polychloroprene, MPs/mL | -0.018 | 0.955 | 1.000 | No | -0.586 | 0.561 |  |
| Motile % - Polyester, MPs/mL | -0.563 | 0.056 | 0.188 | No | -0.872 | 0.065 |  |
| Motile % - Polyethylene, MPs/mL | -0.608 | 0.036 | 0.179 | No | -0.889 | 0.005 |  |
| Motile % - Polypropylene, MPs/mL | 0.000 | 1.000 | 1.000 | No | -0.574 | 0.574 |  |
| Motile % - Polystyrene, MPs/mL | 0.438 | 0.155 | 0.387 | No | -0.211 | 0.819 |  |
| Motile % - Polyamide, MPs/mL | -0.616 | 0.033 | 0.328 | No | -0.892 | -0.006 |  |
| Motile % - Polyimide, MPs/mL | -0.393 | 0.206 | 0.344 | No | -0.798 | 0.257 |  |
| Motile % - Rubber, MPs/mL | 0.014 | 0.965 | 1.000 | No | -0.564 | 0.583 |  |
| Rapid velocity % - Total MPs, MPs/mL | -0.608 | 0.036 | 0.119 | No | -0.889 | 0.005 |  |
| Rapid velocity % - Acrylates, MPs/mL | -0.521 | 0.082 | 0.206 | No | -0.855 | 0.118 |  |
| Rapid velocity % - Polychloroprene, MPs/mL | -0.321 | 0.309 | 0.617 | No | -0.763 | 0.325 |  |
| Rapid velocity % - Polyester, MPs/mL | -0.803 | 0.002 | 0.017 | Yes | -0.952 | -0.341 |  |
| Rapid velocity % - Polyethylene, MPs/mL | -0.755 | 0.005 | 0.023 | Yes | -0.939 | -0.240 |  |
| Rapid velocity % - Polypropylene, MPs/mL | -0.167 | 0.604 | 0.672 | No | -0.678 | 0.454 |  |
| Rapid velocity % - Polystyrene, MPs/mL | 0.037 | 0.908 | 0.908 | No | -0.548 | 0.599 |  |
| Rapid velocity % - Polyamide, MPs/mL | -0.261 | 0.412 | 0.589 | No | -0.731 | 0.377 |  |
| Rapid velocity % - Polyimide, MPs/mL | -0.306 | 0.334 | 0.556 | No | -0.755 | 0.339 |  |
| Rapid velocity % - Rubber, MPs/mL | -0.254 | 0.427 | 0.533 | No | -0.727 | 0.384 |  |
| Medium velocity % - Total MPs, MPs/mL | 0.573 | 0.051 | 0.171 | No | -0.052 | 0.876 |  |
| Medium velocity % - Acrylates, MPs/mL | 0.718 | 0.009 | 0.043 | Yes | 0.170 | 0.927 |  |
| Medium velocity % - Polychloroprene, MPs/mL | 0.450 | 0.142 | 0.237 | No | -0.198 | 0.824 |  |
| Medium velocity % - Polyester, MPs/mL | 0.831 | 0.001 | 0.008 | Yes | 0.408 | 0.960 |  |
| Medium velocity % - Polyethylene, MPs/mL | 0.524 | 0.080 | 0.200 | No | -0.114 | 0.856 |  |
| Medium velocity % - Polypropylene, MPs/mL | -0.036 | 0.911 | 0.911 | No | -0.598 | 0.549 |  |
| Medium velocity % - Polystyrene, MPs/mL | 0.254 | 0.425 | 0.531 | No | -0.383 | 0.728 |  |
| Medium velocity % - Polyamide, MPs/mL | 0.065 | 0.840 | 0.934 | No | -0.529 | 0.617 |  |
| Medium velocity % - Polyimide, MPs/mL | 0.306 | 0.334 | 0.477 | No | -0.339 | 0.755 |  |
| Medium velocity % - Rubber, MPs/mL | 0.465 | 0.128 | 0.256 | No | -0.182 | 0.831 |  |
| Slow velocity % - Total MPs, MPs/mL | 0.601 | 0.039 | 0.129 | No | -0.015 | 0.886 |  |
| Slow velocity % - Acrylates, MPs/mL | 0.493 | 0.103 | 0.259 | No | -0.151 | 0.843 |  |
| Slow velocity % - Polychloroprene, MPs/mL | 0.257 | 0.420 | 0.600 | No | -0.381 | 0.729 |  |
| Slow velocity % - Polyester, MPs/mL | 0.761 | 0.004 | 0.041 | Yes | 0.250 | 0.940 |  |
| Slow velocity % - Polyethylene, MPs/mL | 0.734 | 0.007 | 0.033 | Yes | 0.199 | 0.932 |  |
| Slow velocity % - Polypropylene, MPs/mL | 0.123 | 0.703 | 0.781 | No | -0.487 | 0.653 |  |
| Slow velocity % - Polystyrene, MPs/mL | -0.060 | 0.853 | 0.853 | No | -0.613 | 0.533 |  |
| Slow velocity % - Polyamide, MPs/mL | 0.326 | 0.301 | 0.601 | No | -0.320 | 0.765 |  |
| Slow velocity % - Polyimide, MPs/mL | 0.306 | 0.334 | 0.556 | No | -0.339 | 0.755 |  |
| Slow velocity % - Rubber, MPs/mL | 0.162 | 0.615 | 0.769 | No | -0.458 | 0.676 |  |
| Rapid progressive % - Total MPs, MPs/mL | -0.531 | 0.075 | 0.251 | No | -0.859 | 0.105 |  |
| Rapid progressive % - Acrylates, MPs/mL | -0.380 | 0.223 | 0.557 | No | -0.792 | 0.269 |  |
| Rapid progressive % - Polychloroprene, MPs/mL | -0.321 | 0.309 | 0.617 | No | -0.763 | 0.325 |  |
| Rapid progressive % - Polyester, MPs/mL | -0.789 | 0.002 | 0.023 | Yes | -0.948 | -0.310 |  |
| Rapid progressive % - Polyethylene, MPs/mL | -0.748 | 0.005 | 0.026 | Yes | -0.936 | -0.226 |  |
| Rapid progressive % - Polypropylene, MPs/mL | -0.210 | 0.512 | 0.569 | No | -0.703 | 0.419 |  |
| Rapid progressive % - Polystyrene, MPs/mL | -0.052 | 0.872 | 0.872 | No | -0.608 | 0.538 |  |
| Rapid progressive % - Polyamide, MPs/mL | -0.232 | 0.468 | 0.585 | No | -0.716 | 0.402 |  |
| Rapid progressive % - Polyimide, MPs/mL | -0.306 | 0.334 | 0.556 | No | -0.755 | 0.339 |  |
| Rapid progressive % - Rubber, MPs/mL | -0.261 | 0.413 | 0.591 | No | -0.731 | 0.378 |  |
| Medium progressive % - Total MPs, MPs/mL | 0.545 | 0.067 | 0.167 | No | -0.088 | 0.865 |  |
| Medium progressive % - Acrylates, MPs/mL | 0.655 | 0.021 | 0.208 | No | 0.064 | 0.906 |  |
| Medium progressive % - Polychloroprene, MPs/mL | 0.560 | 0.058 | 0.194 | No | -0.070 | 0.871 |  |
| Medium progressive % - Polyester, MPs/mL | 0.627 | 0.029 | 0.146 | No | 0.021 | 0.896 |  |
| Medium progressive % - Polyethylene, MPs/mL | 0.406 | 0.191 | 0.318 | No | -0.244 | 0.804 |  |
| Medium progressive % - Polypropylene, MPs/mL | 0.138 | 0.669 | 0.744 | No | -0.476 | 0.661 |  |
| Medium progressive % - Polystyrene, MPs/mL | 0.344 | 0.273 | 0.391 | No | -0.304 | 0.774 |  |
| Medium progressive % - Polyamide, MPs/mL | -0.152 | 0.637 | 0.796 | No | -0.670 | 0.465 |  |
| Medium progressive % - Polyimide, MPs/mL | -0.044 | 0.893 | 0.893 | No | -0.603 | 0.544 |  |
| Medium progressive % - Rubber, MPs/mL | 0.521 | 0.082 | 0.165 | No | -0.118 | 0.855 |  |
