# Supplementary material for: Microplastics in the seminal microenvironment of boar semen: associations with sperm motility and antimicrobial susceptibility
Source: Front Vet Sci. 2026 May 26;13:1847076. doi: 10.3389/fvets.2026.1847076 (PMC13271002; doi:10.3389/fvets.2026.1847076)
Supplement: Supplementary file 3 [file Table_3.docx]

Supplementary Material

Table S3. Spearman’s rank correlation coefficients (ρ), two‑tailed p‑values, Benjamini–Hochberg false discovery rate (FDR)‑adjusted p‑values, and 95% confidence intervals (Bonett and Wright method) for correlations between microplastic (MPs) particle size fraction concentrations (MPs/mL) and sperm motility parameters measured by computer‑assisted sperm analysis (CASA) in boar semen samples (n = 12). MPs size distribution categories included <50 µm, 50–100 µm, 100–250 µm, 250–500 µm, 500–1000 µm, and ≥1000 µm; particles that matched the lower limit of each range were assigned to that category. Statistical significance after FDR correction was defined as p(FDR) < 0.05.

|  | Spearman's rho ρ | Significance (2-tailed)  p-value | p-value (FDR) | Significant after FDR (Yes/No) | 95% Confidence Intervals (2-tailed) | |
| --- | --- | --- | --- | --- | --- | --- |
|  |  |  |  |  | **Lower** | **Upper** |
| Motile % - <50µm, MPs/mL | -0.441 | 0.151 | 0.227 | No | -0.820 | 0.208 |
| Motile % - 50-100µm, MPs/mL | -0.134 | 0.678 | 0.678 | No | -0.659 | 0.479 |
| Motile % - 100-250µm, MPs/mL | -0.552 | 0.063 | 0.188 | No | -0.868 | 0.079 |
| Motile % - 250-500µm, MPs/mL | -0.399 | 0.199 | 0.239 | No | -0.801 | 0.251 |
| Motile % - 500-1000µm, MPs/mL | -0.587 | 0.045 | 0.268 | No | -0.881 | 0.034 |
| Motile % - ≥1000µm, MPs/mL | -0.448 | 0.144 | 0.288 | No | -0.824 | 0.200 |
| Rapid velocity % - <50µm, MPs/mL | -0.521 | 0.082 | 0.099 | No | -0.855 | 0.117 |
| Rapid velocity % - 50-100µm, MPs/mL | -0.120 | 0.711 | 0.711 | No | -0.650 | 0.489 |
| Rapid velocity % - 100-250µm, MPs/mL | -0.615 | 0.033 | 0.049 | Yes | -0.892 | -0.005 |
| Rapid velocity % - 250-500µm, MPs/mL | -0.769 | 0.003 | 0.021 | Yes | -0.943 | -0.268 |
| Rapid velocity % - 500-1000µm, MPs/mL | -0.727 | 0.007 | 0.015 | Yes | -0.930 | -0.186 |
| Rapid velocity % - ≥1000µm, MPs/mL | -0.736 | 0.006 | 0.019 | Yes | -0.932 | -0.202 |
| Medium velocity % - <50µm, MPs/mL | 0.521 | 0.082 | 0.123 | No | -0.117 | 0.855 |
| Medium velocity % - 50-100µm, MPs/mL | 0.197 | 0.539 | 0.539 | No | -0.430 | 0.696 |
| Medium velocity % - 100-250µm, MPs/mL | 0.503 | 0.095 | 0.114 | No | -0.139 | 0.848 |
| Medium velocity % - 250-500µm, MPs/mL | 0.741 | 0.006 | 0.035 | Yes | 0.212 | 0.934 |
| Medium velocity % - 500-1000µm, MPs/mL | 0.587 | 0.045 | 0.089 | No | -0.034 | 0.881 |
| Medium velocity % - ≥1000µm, MPs/mL | 0.690 | 0.013 | 0.039 | Yes | 0.120 | 0.918 |
| Slow velocity % - <50µm, MPs/mL | 0.521 | 0.082 | 0.099 | No | -0.117 | 0.855 |
| Slow velocity % - 50-100µm, MPs/mL | 0.085 | 0.794 | 0.794 | No | -0.515 | 0.629 |
| Slow velocity % - 100-250µm, MPs/mL | 0.643 | 0.024 | 0.036 | Yes | 0.046 | 0.902 |
| Slow velocity % - 250-500µm, MPs/mL | 0.762 | 0.004 | 0.024 | Yes | 0.254 | 0.941 |
| Slow velocity % - 500-1000µm, MPs/mL | 0.692 | 0.013 | 0.038 | Yes | 0.124 | 0.919 |
| Slow velocity % - ≥1000µm, MPs/mL | 0.683 | 0.014 | 0.029 | Yes | 0.109 | 0.915 |
| Rapid progressive % - <50µm, MPs/mL | -0.521 | 0.082 | 0.099 | No | -0.855 | 0.117 |
| Rapid progressive % - 50-100µm, MPs/mL | -0.141 | 0.662 | 0.662 | No | -0.663 | 0.474 |
| Rapid progressive % - 100-250µm, MPs/mL | -0.524 | 0.080 | 0.120 | No | -0.856 | 0.114 |
| Rapid progressive % - 250-500µm, MPs/mL | -0.706 | 0.010 | 0.031 | Yes | -0.923 | -0.148 |
| Rapid progressive % - 500-1000µm, MPs/mL | -0.699 | 0.011 | 0.023 | Yes | -0.921 | -0.136 |
| Rapid progressive % - ≥1000µm, MPs/mL | -0.739 | 0.006 | 0.036 | Yes | -0.934 | -0.208 |
| Medium progressive % - <50µm, MPs/mL | 0.355 | 0.258 | 0.309 | No | -0.294 | 0.780 |
| Medium progressive % - 50-100µm, MPs/mL | 0.282 | 0.375 | 0.375 | No | -0.360 | 0.742 |
| Medium progressive % - 100-250µm, MPs/mL | 0.406 | 0.191 | 0.382 | No | -0.244 | 0.804 |
| Medium progressive % - 250-500µm, MPs/mL | 0.615 | 0.033 | 0.199 | No | 0.005 | 0.892 |
| Medium progressive % - 500-1000µm, MPs/mL | 0.406 | 0.191 | 0.286 | No | -0.244 | 0.804 |
| Medium progressive % - ≥1000µm, MPs/mL | 0.518 | 0.084 | 0.253 | No | -0.121 | 0.854 |
