# Supplementary material for: Microplastics in the seminal microenvironment of boar semen: associations with sperm motility and antimicrobial susceptibility
Source: Front Vet Sci. 2026 May 26;13:1847076. doi: 10.3389/fvets.2026.1847076 (PMC13271002; doi:10.3389/fvets.2026.1847076)
Supplement: Supplementary file 4 [file Table_4.docx]

Supplementary Material

Table S4. Spearman’s rank correlation coefficients (ρ), two‑tailed significance levels (p‑values), Benjamini–Hochberg false discovery rate (FDR)‑adjusted p‑values, and 95% confidence intervals (Bonett and Wright method) for correlations between microplastic (MPs) concentrations (total, polymer‑specific, and particle size‑specific counts, MPs/mL) and sperm morphology parameters (tail defects, head defects, midpiece defects, and total pathological spermatozoa, %) in boar semen samples (n = 12). Statistical significance after FDR correction was defined as p(FDR) < 0.05.

|  | Spearman's rho ρ | Significance (2-tailed)  p-value | p-value (FDR) | Significant after FDR (Yes/No) | 95% Confidence Intervals (2-tailed) | |
| --- | --- | --- | --- | --- | --- | --- |
|  |  |  |  |  | **Lower** | **Upper** |
| Tail defects % - Total MPs, MPs/mL | 0.477 | 0.117 | 0.336 | No | -0.169 | 0.836 |
| Tail defects % - Acrylates, MPs/mL | 0.261 | 0.412 | 0.470 | No | -0.377 | 0.732 |
| Tail defects % - Polychloroprene, MPs/mL | 0.378 | 0.226 | 0.449 | No | -0.272 | 0.791 |
| Tail defects % - Polyester, MPs/mL | 0.481 | 0.114 | 0.336 | No | -0.165 | 0.838 |
| Tail defects % - Polyethylene, MPs/mL | 0.228 | 0.476 | 0.508 | No | -0.405 | 0.713 |
| Tail defects % - Polypropylene, MPs/mL | -0.287 | 0.365 | 0.449 | No | -0.745 | 0.355 |
| Tail defects % - Polystyrene, MPs/mL | -0.298 | 0.346 | 0.449 | No | -0.751 | 0.345 |
| Tail defects % - Polyamide, MPs/mL | 0.386 | 0.216 | 0.449 | No | -0.264 | 0.795 |
| Tail defects % - Polyimide, MPs/mL | 0.482 | 0.112 | 0.336 | No | -0.163 | 0.838 |
| Tail defects % - Rubber, MPs/mL | 0.187 | 0.560 | 0.560 | No | -0.438 | 0.690 |
| Tail defects % - <50µm, MPs/mL | 0.326 | 0.301 | 0.449 | No | -0.320 | 0.765 |
| Tail defects % - 50-100µm, MPs/mL | 0.583 | 0.047 | 0.336 | No | -0.040 | 0.880 |
| Tail defects % - 100-250µm, MPs/mL | 0.467 | 0.126 | 0.336 | No | -0.180 | 0.832 |
| Tail defects % - 250-500µm, MPs/mL | 0.288 | 0.364 | 0.449 | No | -0.355 | 0.746 |
| Tail defects % - 500-1000µm, MPs/mL | 0.509 | 0.091 | 0.336 | No | -0.132 | 0.850 |
| Tail defects % - ≥1000µm, MPs/mL | 0.346 | 0.270 | 0.449 | No | -0.302 | 0.775 |
| Head defects % - Total MPs, MPs/mL | 0.169 | 0.600 | 0.959 | No | -0.452 | 0.680 |
| Head defects % - Acrylates, MPs/mL | -0.015 | 0.963 | 0.963 | No | -0.584 | 0.564 |
| Head defects % - Polychloroprene, MPs/mL | 0.020 | 0.952 | 0.963 | No | -0.561 | 0.587 |
| Head defects % - Polyester, MPs/mL | 0.060 | 0.852 | 0.963 | No | -0.532 | 0.613 |
| Head defects % - Polyethylene, MPs/mL | 0.116 | 0.719 | 0.959 | No | -0.492 | 0.648 |
| Head defects % - Polypropylene, MPs/mL | 0.260 | 0.414 | 0.959 | No | -0.378 | 0.731 |
| Head defects % - Polystyrene, MPs/mL | -0.208 | 0.516 | 0.959 | No | -0.702 | 0.421 |
| Head defects % - Polyamide, MPs/mL | -0.187 | 0.562 | 0.959 | No | -0.690 | 0.438 |
| Head defects % - Polyimide, MPs/mL | 0.281 | 0.377 | 0.959 | No | -0.361 | 0.742 |
| Head defects % - Rubber, MPs/mL | 0.445 | 0.147 | 0.959 | No | -0.203 | 0.822 |
| Head defects % - <50µm, MPs/mL | -0.035 | 0.915 | 0.963 | No | -0.597 | 0.550 |
| Head defects % - 50-100µm, MPs/mL | 0.381 | 0.222 | 0.959 | No | -0.268 | 0.792 |
| Head defects % - 100-250µm, MPs/mL | 0.169 | 0.600 | 0.959 | No | -0.452 | 0.680 |
| Head defects % - 250-500µm, MPs/mL | 0.127 | 0.693 | 0.959 | No | -0.484 | 0.655 |
| Head defects % - 500-1000µm, MPs/mL | -0.206 | 0.520 | 0.959 | No | -0.701 | 0.423 |
| Head defects % - ≥1000µm, MPs/mL | -0.126 | 0.697 | 0.959 | No | -0.654 | 0.485 |
| Midpiece defects % - Total MPs, MPs/mL | -0.182 | 0.572 | 0.983 | No | -0.687 | 0.442 |
| Midpiece defects % - Acrylates, MPs/mL | 0.106 | 0.744 | 0.983 | No | -0.500 | 0.642 |
| Midpiece defects % - Polychloroprene, MPs/mL | -0.459 | 0.133 | 0.660 | No | -0.828 | 0.189 |
| Midpiece defects % - Polyester, MPs/mL | -0.028 | 0.931 | 0.983 | No | -0.593 | 0.555 |
| Midpiece defects % - Polyethylene, MPs/mL | -0.091 | 0.779 | 0.983 | No | -0.633 | 0.511 |
| Midpiece defects % - Polypropylene, MPs/mL | -0.522 | 0.082 | 0.660 | No | -0.855 | 0.117 |
| Midpiece defects % - Polystyrene, MPs/mL | -0.516 | 0.086 | 0.660 | No | -0.853 | 0.124 |
| Midpiece defects % - Polyamide, MPs/mL | 0.428 | 0.165 | 0.660 | No | -0.222 | 0.814 |
| Midpiece defects % - Polyimide, MPs/mL | 0.393 | 0.206 | 0.660 | No | -0.257 | 0.798 |
| Midpiece defects % - Rubber, MPs/mL | -0.077 | 0.811 | 0.983 | No | -0.624 | 0.520 |
| Midpiece defects % - <50µm, MPs/mL | -0.048 | 0.881 | 0.983 | No | -0.606 | 0.541 |
| Midpiece defects % - 50-100µm, MPs/mL | -0.246 | 0.440 | 0.983 | No | -0.723 | 0.390 |
| Midpiece defects % - 100-250µm, MPs/mL | -0.007 | 0.983 | 0.983 | No | -0.579 | 0.569 |
| Midpiece defects % - 250-500µm, MPs/mL | -0.105 | 0.746 | 0.983 | No | -0.641 | 0.500 |
| Midpiece defects % - 500-1000µm, MPs/mL | -0.042 | 0.897 | 0.983 | No | -0.602 | 0.545 |
| Midpiece defects % - ≥1000µm, MPs/mL | -0.196 | 0.541 | 0.983 | No | -0.695 | 0.431 |
| Total pathological sperm % - Total MPs, MPs/mL | 0.173 | 0.591 | 0.957 | No | -0.449 | 0.682 |
| Total pathological sperm % - Acrylates, MPs/mL | 0.146 | 0.652 | 0.957 | No | -0.470 | 0.666 |
| Total pathological sperm % - Polychloroprene, MPs/mL | -0.079 | 0.808 | 0.957 | No | -0.625 | 0.519 |
| Total pathological sperm % - Polyester, MPs/mL | 0.146 | 0.652 | 0.957 | No | -0.470 | 0.666 |
| Total pathological sperm % - Polyethylene, MPs/mL | 0.060 | 0.853 | 0.957 | No | -0.533 | 0.613 |
| Total pathological sperm % - Polypropylene, MPs/mL | -0.369 | 0.237 | 0.949 | No | -0.787 | 0.280 |
| Total pathological sperm % - Polystyrene, MPs/mL | -0.496 | 0.101 | 0.949 | No | -0.844 | 0.147 |
| Total pathological sperm % - Polyamide, MPs/mL | 0.443 | 0.150 | 0.949 | No | -0.206 | 0.821 |
| Total pathological sperm % - Polyimide, MPs/mL | 0.397 | 0.202 | 0.949 | No | -0.253 | 0.800 |
| Total pathological sperm % - Rubber, MPs/mL | 0.085 | 0.792 | 0.957 | No | -0.515 | 0.629 |
| Total pathological sperm % - <50µm, MPs/mL | 0.022 | 0.947 | 0.957 | No | -0.559 | 0.588 |
| Total pathological sperm % - 50-100µm, MPs/mL | 0.199 | 0.535 | 0.957 | No | -0.429 | 0.697 |
| Total pathological sperm % - 100-250µm, MPs/mL | 0.265 | 0.406 | 0.957 | No | -0.375 | 0.733 |
| Total pathological sperm % - 250-500µm, MPs/mL | 0.074 | 0.819 | 0.957 | No | -0.523 | 0.622 |
| Total pathological sperm % - 500-1000µm, MPs/mL | 0.194 | 0.546 | 0.957 | No | -0.432 | 0.694 |
| Total pathological sperm % - ≥1000µm, MPs/mL | 0.018 | 0.957 | 0.957 | No | -0.562 | 0.586 |
