# Supplementary material for: Microplastics in the seminal microenvironment of boar semen: associations with sperm motility and antimicrobial susceptibility
Source: Front Vet Sci. 2026 May 26;13:1847076. doi: 10.3389/fvets.2026.1847076 (PMC13271002; doi:10.3389/fvets.2026.1847076)
Supplement: Supplementary file 5 [file Table_5.docx]

Supplementary Material

Table S5. Spearman’s rank correlation coefficients (ρ), two‑tailed significance levels (p-values), Benjamini–Hochberg false discovery rate (FDR)–adjusted p‑values, and 95% confidence intervals (Bonett and Wright method) for correlations between microplastic (MPs) concentrations (total and polymer‑specific particle counts, MPs/mL) and minimum inhibitory concentration (MIC) values of bacterial isolates recovered from boar semen samples. Correlation analyses were performed separately for *Escherichia coli*, *Pseudomonas aeruginosa* and *Staphylococcus equorum* bacterial species–antibiotic combination. Statistical significance after FDR correction was defined as p(FDR) < 0.05.

|  | Spearman's rho ρ | Significance (2-tailed)  p-value | p-value (FDR) | Significant after FDR (Yes/No) | 95% Confidence Intervals (2-tailed) | | |
| --- | --- | --- | --- | --- | --- | --- | --- |
|  |  |  |  |  | **Lower** | **Upper** | |
| *E. coli* Gentamicin MIC - Total MPs, MPs/mL | -0.488 | 0.183 | 0.915 | No | -0.881 | 0.304 |  |
| *E. coli* Gentamicin MIC - Acrylates, MPs/mL | 0.205 | 0.597 | 0.931 | No | -0.538 | 0.768 |  |
| *E. coli* Gentamicin MIC - Polychloroprene, MPs/mL | -0.571 | 0.109 | 0.915 | No | -0.907 | 0.211 |  |
| *E. coli* Gentamicin MIC - Polyester, MPs/mL | -0.080 | 0.838 | 0.931 | No | -0.707 | 0.618 |  |
| *E. coli* Gentamicin MIC - Polyethylene, MPs/mL | 0.089 | 0.821 | 0.931 | No | -0.612 | 0.712 |  |
| *E. coli* Gentamicin MIC - Polypropylene, MPs/mL | 0.088 | 0.822 | 0.931 | No | -0.613 | 0.711 |  |
| *E. coli* Gentamicin MIC - Polystyrene, MPs/mL | -0.185 | 0.635 | 0.931 | No | -0.759 | 0.551 |  |
| *E. coli* Gentamicin MIC - Polyamide, MPs/mL | -0.374 | 0.321 | 0.931 | No | -0.840 | 0.409 |  |
| *E. coli* Gentamicin MIC - Polyimide, MPs/mL | 0.291 | 0.447 | 0.931 | No | -0.475 | 0.807 |  |
| *E. coli* Gentamicin MIC - Rubber, MPs/mL | 0.004 | 0.991 | 0.991 | No | -0.662 | 0.667 |  |
| *E. coli* Streptomycin MIC - Total MPs, MPs/mL | 0.179 | 0.645 | 0.907 | No | -0.555 | 0.756 |  |
| *E. coli* Streptomycin MIC - Acrylates, MPs/mL | -0.261 | 0.498 | 0.907 | No | -0.793 | 0.498 |  |
| *E. coli* Streptomycin MIC - Polychloroprene, MPs/mL | -0.047 | 0.905 | 1.000 | No | -0.690 | 0.637 |  |
| *E. coli* Streptomycin MIC - Polyester, MPs/mL | 0.204 | 0.598 | 0.907 | No | -0.538 | 0.768 |  |
| *E. coli* Streptomycin MIC - Polyethylene, MPs/mL | 0.604 | 0.085 | 0.424 | No | -0.169 | 0.917 |  |
| *E. coli* Streptomycin MIC - Polypropylene, MPs/mL | 0.471 | 0.200 | 0.668 | No | -0.320 | 0.875 |  |
| *E. coli* Streptomycin MIC - Polystyrene, MPs/mL | -0.821 | 0.007 | 0.067 | No | -0.969 | -0.229 |  |
| *E. coli* Streptomycin MIC - Polyamide, MPs/mL | 0.205 | 0.596 | 0.907 | No | -0.537 | 0.768 |  |
| *E. coli* Streptomycin MIC - Polyimide, MPs/mL | 0.000 | 1.000 | 1.000 | No | -0.664 | 0.664 |  |
| *E. coli* Streptomycin MIC - Rubber, MPs/mL | 0.137 | 0.726 | 0.907 | No | -0.583 | 0.736 |  |
| *E. coli* Spectinomycin MIC - Total MPs, MPs/mL | 0.205 | 0.596 | 0.809 | No | -0.537 | 0.769 |  |
| *E. coli* Spectinomycin MIC - Acrylates, MPs/mL | 0.790 | 0.011 | 0.113 | No | 0.153 | 0.963 |  |
| *E. coli* Spectinomycin MIC - Polychloroprene, MPs/mL | 0.082 | 0.834 | 0.861 | No | -0.617 | 0.708 |  |
| *E. coli* Spectinomycin MIC - Polyester, MPs/mL | 0.624 | 0.072 | 0.362 | No | -0.142 | 0.923 |  |
| *E. coli* Spectinomycin MIC - Polyethylene, MPs/mL | 0.205 | 0.596 | 0.809 | No | -0.537 | 0.769 |  |
| *E. coli* Spectinomycin MIC - Polypropylene, MPs/mL | -0.357 | 0.345 | 0.809 | No | -0.833 | 0.423 |  |
| *E. coli* Spectinomycin MIC - Polystyrene, MPs/mL | 0.178 | 0.647 | 0.809 | No | -0.556 | 0.756 |  |
| *E. coli* Spectinomycin MIC - Polyamide, MPs/mL | -0.069 | 0.861 | 0.861 | No | -0.701 | 0.624 |  |
| *E. coli* Spectinomycin MIC - Polyimide, MPs/mL | 0.562 | 0.115 | 0.384 | No | -0.222 | 0.905 |  |
| *E. coli* Spectinomycin MIC - Rubber, MPs/mL | 0.206 | 0.595 | 0.809 | No | -0.537 | 0.769 |  |
| *E. coli* Amoxicillin MIC - Total MPs, MPs/mL | 0.202 | 0.603 | 0.749 | No | -0.540 | 0.767 |  |
| *E. coli* Amoxicillin MIC - Acrylates, MPs/mL | -0.189 | 0.626 | 0.749 | No | -0.761 | 0.548 |  |
| *E. coli* Amoxicillin MIC - Polychloroprene, MPs/mL | -0.239 | 0.536 | 0.749 | No | -0.784 | 0.514 |  |
| *E. coli* Amoxicillin MIC - Polyester, MPs/mL | 0.294 | 0.443 | 0.749 | No | -0.474 | 0.807 |  |
| *E. coli* Amoxicillin MIC - Polyethylene, MPs/mL | 0.615 | 0.078 | 0.260 | No | -0.155 | 0.920 |  |
| *E. coli* Amoxicillin MIC - Polypropylene, MPs/mL | 0.125 | 0.749 | 0.749 | No | -0.590 | 0.730 |  |
| *E. coli* Amoxicillin MIC - Polystyrene, MPs/mL | -0.915 | 0.001 | 0.006 | Yes | -0.987 | -0.538 |  |
| *E. coli* Amoxicillin MIC - Polyamide, MPs/mL | 0.617 | 0.077 | 0.260 | No | -0.151 | 0.921 |  |
| *E. coli* Amoxicillin MIC - Polyimide, MPs/mL | 0.302 | 0.430 | 0.749 | No | -0.468 | 0.811 |  |
| *E. coli* Amoxicillin MIC - Rubber, MPs/mL | -0.161 | 0.679 | 0.749 | No | -0.748 | 0.567 |  |
| *P. aeruginosa* Gentamicin MIC - Total MPs, MPs/mL | -0.899 | 0.015 | 0.069 | No | -0.993 | -0.124 |  |
| *P. aeruginosa* Gentamicin MIC - Acrylates, MPs/mL | -0.609 | 0.200 | 0.359 | No | -0.959 | 0.482 |  |
| *P. aeruginosa* Gentamicin MIC - Polychloroprene, MPs/mL | -0.857 | 0.029 | 0.087 | No | -0.989 | 0.040 |  |
| *P. aeruginosa* Gentamicin MIC - Polyester, MPs/mL | -0.897 | 0.015 | 0.069 | No | -0.993 | -0.116 |  |
| *P. aeruginosa* Gentamicin MIC - Polyethylene, MPs/mL | -0.406 | 0.425 | 0.546 | No | -0.923 | 0.633 |  |
| *P. aeruginosa* Gentamicin MIC - Polypropylene, MPs/mL | 0.277 | 0.595 | 0.669 | No | -0.701 | 0.893 |  |
| *P. aeruginosa* Gentamicin MIC - Polystyrene, MPs/mL | -0.154 | 0.771 | 0.771 | No | -0.860 | 0.754 |  |
| *P. aeruginosa* Gentamicin MIC - Polyamide, MPs/mL | -0.493 | 0.321 | 0.481 | No | -0.940 | 0.577 |  |
| *P. aeruginosa* Gentamicin MIC - Polyimide, MPs/mL | ND | ND | ND | ND | ND | ND |  |
| *P. aeruginosa* Gentamicin MIC - Rubber, MPs/mL | -0.770 | 0.073 | 0.165 | No | -0.980 | 0.262 |  |
| *P. aeruginosa* Streptomycin MIC - Total MPs, MPs/mL | -0.618 | 0.191 | 0.430 | No | -0.961 | 0.472 |  |
| *P. aeruginosa* Streptomycin MIC - Acrylates, MPs/mL | -0.265 | 0.612 | 0.695 | No | -0.890 | 0.706 |  |
| *P. aeruginosa* Streptomycin MIC - Polychloroprene, MPs/mL | -0.870 | 0.024 | 0.217 | No | -0.990 | -0.006 |  |
| *P. aeruginosa* Streptomycin MIC - Polyester, MPs/mL | -0.746 | 0.088 | 0.398 | No | -0.978 | 0.305 |  |
| *P. aeruginosa* Streptomycin MIC - Polyethylene, MPs/mL | -0.206 | 0.695 | 0.695 | No | -0.875 | 0.733 |  |
| *P. aeruginosa* Streptomycin MIC - Polypropylene, MPs/mL | 0.219 | 0.677 | 0.695 | No | -0.727 | 0.878 |  |
| *P. aeruginosa* Streptomycin MIC - Polystyrene, MPs/mL | -0.375 | 0.464 | 0.695 | No | -0.916 | 0.651 |  |
| *P. aeruginosa* Streptomycin MIC - Polyamide, MPs/mL | -0.250 | 0.633 | 0.695 | No | -0.886 | 0.713 |  |
| *P. aeruginosa* Streptomycin MIC - Polyimide, MPs/mL | ND | ND | ND | ND | ND | ND |  |
| *P. aeruginosa* Streptomycin MIC - Rubber, MPs/mL | -0.657 | 0.157 | 0.430 | No | -0.966 | 0.431 |  |
| *P. aeruginosa* Spectinomycin MIC - Total MPs, MPs/mL | -0.679 | 0.138 | 0.351 | No | -0.969 | 0.404 |  |
| *P. aeruginosa* Spectinomycin MIC - Acrylates, MPs/mL | -0.494 | 0.320 | 0.457 | No | -0.940 | 0.577 |  |
| *P. aeruginosa* Spectinomycin MIC - Polychloroprene, MPs/mL | -0.657 | 0.156 | 0.351 | No | -0.966 | 0.430 |  |
| *P. aeruginosa* Spectinomycin MIC - Polyester, MPs/mL | -0.892 | 0.017 | 0.085 | No | -0.992 | -0.095 |  |
| *P. aeruginosa* Spectinomycin MIC - Polyethylene, MPs/mL | -0.463 | 0.355 | 0.457 | No | -0.934 | 0.598 |  |
| *P. aeruginosa* Spectinomycin MIC - Polypropylene, MPs/mL | 0.098 | 0.853 | 0.853 | No | -0.776 | 0.843 |  |
| *P. aeruginosa* Spectinomycin MIC - Polystyrene, MPs/mL | -0.557 | 0.250 | 0.451 | No | -0.951 | 0.528 |  |
| *P. aeruginosa* Spectinomycin MIC - Polyamide, MPs/mL | -0.393 | 0.440 | 0.495 | No | -0.920 | 0.640 |  |
| *P. aeruginosa* Spectinomycin MIC - Polyimide, MPs/mL | ND | ND | ND | ND | ND | ND |  |
| *P. aeruginosa* Spectinomycin MIC - Rubber, MPs/mL | -0.885 | 0.019 | 0.085 | No | -0.992 | -0.065 |  |
| *S. equorum* Gentamicin MIC - Total MPs, MPs/mL | -0.408 | 0.363 | 0.740 | No | -0.896 | 0.527 |  |
| *S. equorum* Gentamicin MIC - Acrylates, MPs/mL | -0.515 | 0.237 | 0.740 | No | -0.924 | 0.441 |  |
| *S. equorum* Gentamicin MIC - Polychloroprene, MPs/mL | -0.338 | 0.459 | 0.740 | No | -0.876 | 0.576 |  |
| *S. equorum* Gentamicin MIC - Polyester, MPs/mL | -0.424 | 0.344 | 0.740 | No | -0.901 | 0.516 |  |
| *S. equorum* Gentamicin MIC - Polyethylene, MPs/mL | -0.408 | 0.363 | 0.740 | No | -0.896 | 0.527 |  |
| *S. equorum* Gentamicin MIC - Polypropylene, MPs/mL | 0.212 | 0.648 | 0.740 | No | -0.650 | 0.835 |  |
| *S. equorum* Gentamicin MIC - Polystyrene, MPs/mL | -0.104 | 0.825 | 0.825 | No | -0.796 | 0.706 |  |
| *S. equorum* Gentamicin MIC - Polyamide, MPs/mL | -0.255 | 0.582 | 0.740 | No | -0.850 | 0.626 |  |
| *S. equorum* Gentamicin MIC - Polyimide, MPs/mL | ND | ND | ND | ND | ND | ND |  |
| *S. equorum* Gentamicin MIC - Rubber, MPs/mL | -0.206 | 0.658 | 0.740 | No | -0.833 | 0.653 |  |
| *S. equorum* Streptomycin MIC - Total MPs, MPs/mL | -0.643 | 0.119 | 0.208 | No | -0.951 | 0.304 |  |
| *S. equorum* Streptomycin MIC - Acrylates, MPs/mL | -0.721 | 0.068 | 0.208 | No | -0.965 | 0.188 |  |
| *S. equorum* Streptomycin MIC - Polychloroprene, MPs/mL | -0.374 | 0.408 | 0.459 | No | -0.887 | 0.551 |  |
| *S. equorum* Streptomycin MIC - Polyester, MPs/mL | -0.704 | 0.077 | 0.208 | No | -0.962 | 0.216 |  |
| *S. equorum* Streptomycin MIC - Polyethylene, MPs/mL | -0.679 | 0.094 | 0.208 | No | -0.957 | 0.255 |  |
| *S. equorum* Streptomycin MIC - Polypropylene, MPs/mL | -0.148 | 0.751 | 0.751 | No | -0.813 | 0.684 |  |
| *S. equorum* Streptomycin MIC - Polystyrene, MPs/mL | -0.618 | 0.139 | 0.208 | No | -0.946 | 0.334 |  |
| *S. equorum* Streptomycin MIC - Polyamide, MPs/mL | -0.401 | 0.373 | 0.459 | No | -0.894 | 0.533 |  |
| *S. equorum* Streptomycin MIC - Polyimide, MPs/mL | ND | ND | ND | ND | ND | ND |  |
| *S. equorum* Streptomycin MIC - Rubber, MPs/mL | -0.811 | 0.027 | 0.208 | No | -0.978 | 0.000 |  |
| *S. equorum* Spectinomycin MIC - Total MPs, MPs/mL | -0.257 | 0.578 | 0.650 | No | -0.851 | 0.625 |  |
| *S. equorum* Spectinomycin MIC - Acrylates, MPs/mL | -0.194 | 0.676 | 0.676 | No | -0.829 | 0.660 |  |
| *S. equorum* Spectinomycin MIC - Polychloroprene, MPs/mL | -0.354 | 0.435 | 0.581 | No | -0.881 | 0.565 |  |
| *S. equorum* Spectinomycin MIC - Polyester, MPs/mL | -0.343 | 0.452 | 0.581 | No | -0.878 | 0.572 |  |
| *S. equorum* Spectinomycin MIC - Polyethylene, MPs/mL | -0.587 | 0.166 | 0.497 | No | -0.940 | 0.369 |  |
| *S. equorum* Spectinomycin MIC - Polypropylene, MPs/mL | -0.933 | 0.002 | 0.019 | Yes | -0.993 | -0.470 |  |
| *S. equorum* Spectinomycin MIC - Polystyrene, MPs/mL | -0.907 | 0.005 | 0.022 | Yes | -0.990 | -0.331 |  |
| *S. equorum* Spectinomycin MIC - Polyamide, MPs/mL | 0.527 | 0.225 | 0.506 | No | -0.430 | 0.926 |  |
| *S. equorum* Spectinomycin MIC - Polyimide, MPs/mL | ND | ND | ND | ND | ND | ND |  |
| *S. equorum* Spectinomycin MIC - Rubber, MPs/mL | -0.389 | 0.388 | 0.581 | No | -0.891 | 0.541 |  |
| *S. equorum* Amoxicillin MIC - Total MPs, MPs/mL | -0.551 | 0.200 | 0.450 | No | -0.932 | 0.407 |  |
| *S. equorum* Amoxicillin MIC - Acrylates, MPs/mL | -0.732 | 0.062 | 0.450 | No | -0.966 | 0.170 |  |
| *S. equorum* Amoxicillin MIC - Polychloroprene, MPs/mL | -0.294 | 0.523 | 0.672 | No | -0.863 | 0.603 |  |
| *S. equorum* Amoxicillin MIC - Polyester, MPs/mL | -0.343 | 0.452 | 0.672 | No | -0.878 | 0.572 |  |
| *S. equorum* Amoxicillin MIC - Polyethylene, MPs/mL | -0.404 | 0.369 | 0.664 | No | -0.895 | 0.531 |  |
| *S. equorum* Amoxicillin MIC - Polypropylene, MPs/mL | -0.095 | 0.839 | 0.884 | No | -0.792 | 0.710 |  |
| *S. equorum* Amoxicillin MIC - Polystyrene, MPs/mL | -0.589 | 0.164 | 0.450 | No | -0.940 | 0.368 |  |
| *S. equorum* Amoxicillin MIC - Polyamide, MPs/mL | -0.069 | 0.884 | 0.884 | No | -0.782 | 0.722 |  |
| *S. equorum* Amoxicillin MIC - Polyimide, MPs/mL | ND | ND | ND | ND | ND | ND |  |
| *S. equorum* Amoxicillin MIC - Rubber, MPs/mL | -0.611 | 0.145 | 0.450 | No | -0.945 | 0.342 |  |
| *S. equorum* Penicillin MIC - Total MPs, MPs/mL | -0.148 | 0.751 | 1.000 | No | -0.813 | 0.684 |  |
| *S. equorum* Penicillin MIC - Acrylates, MPs/mL | -0.561 | 0.190 | 0.856 | No | -0.934 | 0.397 |  |
| *S. equorum* Penicillin MIC - Polychloroprene, MPs/mL | 0.000 | 1.000 | 1.000 | No | -0.753 | 0.753 |  |
| *S. equorum* Penicillin MIC - Polyester, MPs/mL | -0.231 | 0.619 | 1.000 | No | -0.842 | 0.640 |  |
| *S. equorum* Penicillin MIC - Polyethylene, MPs/mL | -0.408 | 0.364 | 0.919 | No | -0.896 | 0.528 |  |
| *S. equorum* Penicillin MIC - Polypropylene, MPs/mL | -0.077 | 0.870 | 1.000 | No | -0.785 | 0.718 |  |
| *S. equorum* Penicillin MIC - Polystyrene, MPs/mL | -0.623 | 0.135 | 0.856 | No | -0.947 | 0.329 |  |
| *S. equorum* Penicillin MIC - Polyamide, MPs/mL | -0.046 | 0.922 | 1.000 | No | -0.773 | 0.733 |  |
| *S. equorum* Penicillin MIC - Polyimide, MPs/mL | ND | ND | ND | ND | ND | ND |  |
| *S. equorum* Penicillin MIC - Rubber, MPs/mL | -0.374 | 0.409 | 0.919 | No | -0.887 | 0.552 |  |

ND – not determined. Correlations were not calculated for specific polymer types in samples where the corresponding MPs particles were not detected, as the absence of values did not allow statistically meaningful correlation analysis.
